# Supplementary material for: A vital sign-based prediction algorithm for differentiating COVID-19 versus seasonal influenza in hospitalized patients
Source: NPJ Digit Med. 2021 Jun 4;4:95. doi: 10.1038/s41746-021-00467-8 (PMC8178379; doi:10.1038/s41746-021-00467-8)
Supplement: Supplementary file 1 — Supplementary Information [file 41746_2021_467_MOESM1_ESM.pdf]

Supplemental Information

Supplemental Figures

Supplementary Figure 1. Flowchart showing the study outline and details of the WVU and TrinetX COVID-19 positive, -negative and influenza patient subgroups of considered for this study.

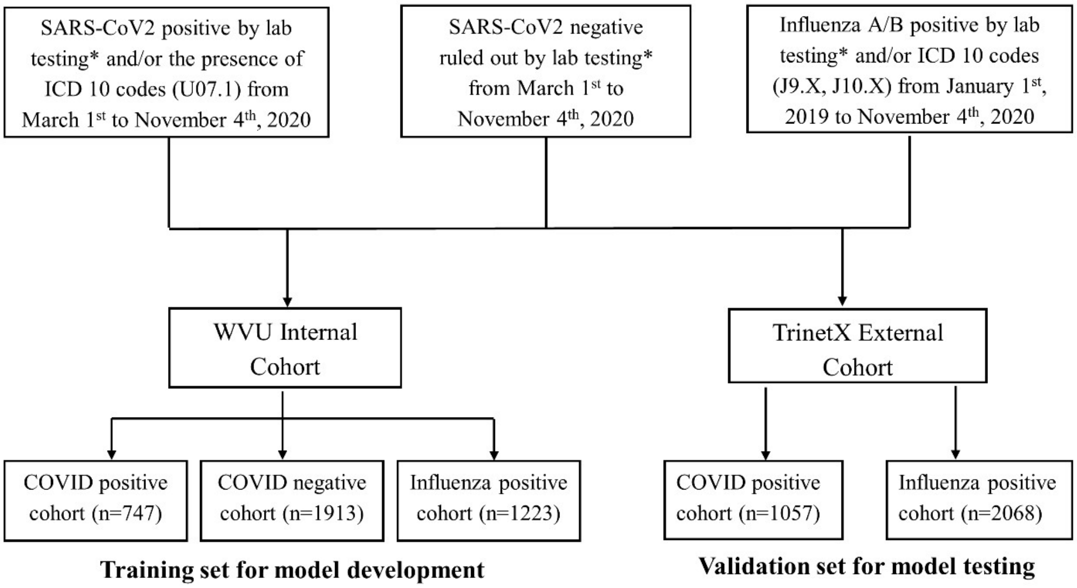

** Confirmed diagnostic testing data is only available for WVU cohort.*

Supplementary Figure 2. SHAP summary plot of the multi-class classifier model that can predict patients that could test positive for Influenza, COVID-19 positive or -negative.

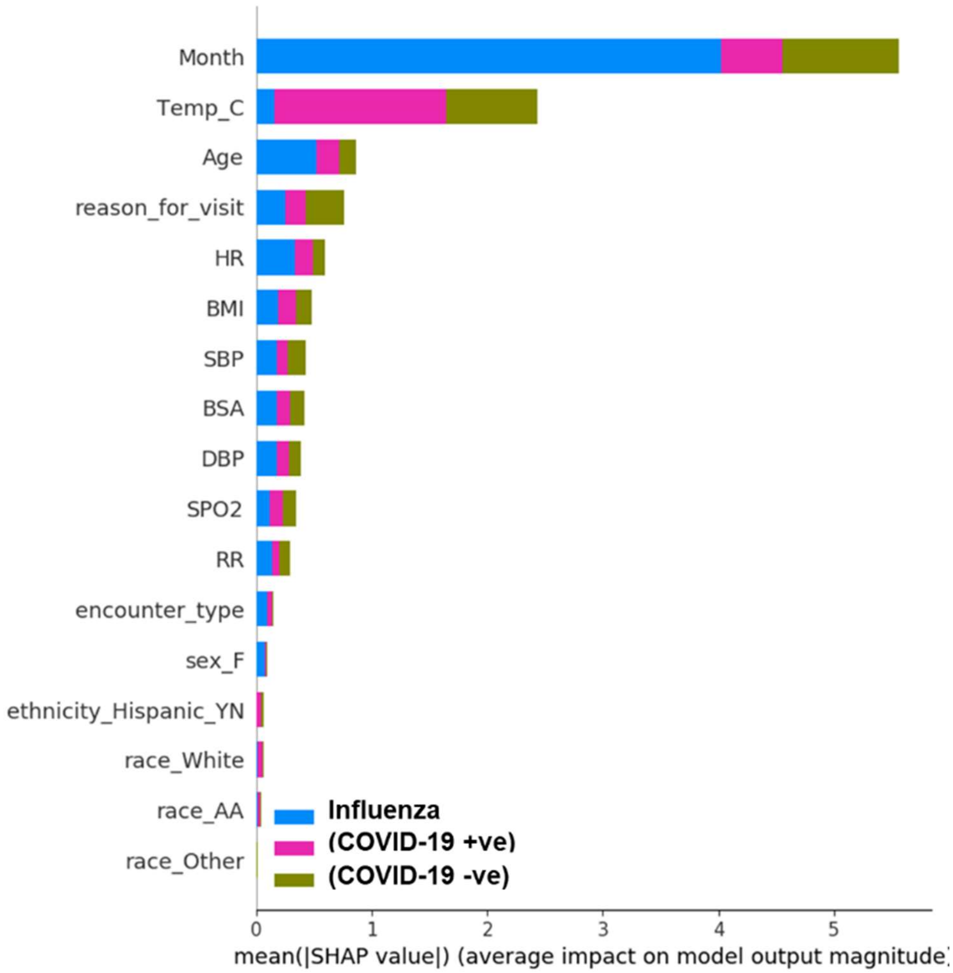

Supplementary Figure 3. The Precision-Recall Curve for the COVID-19-positive vs -negative XGBoost model.

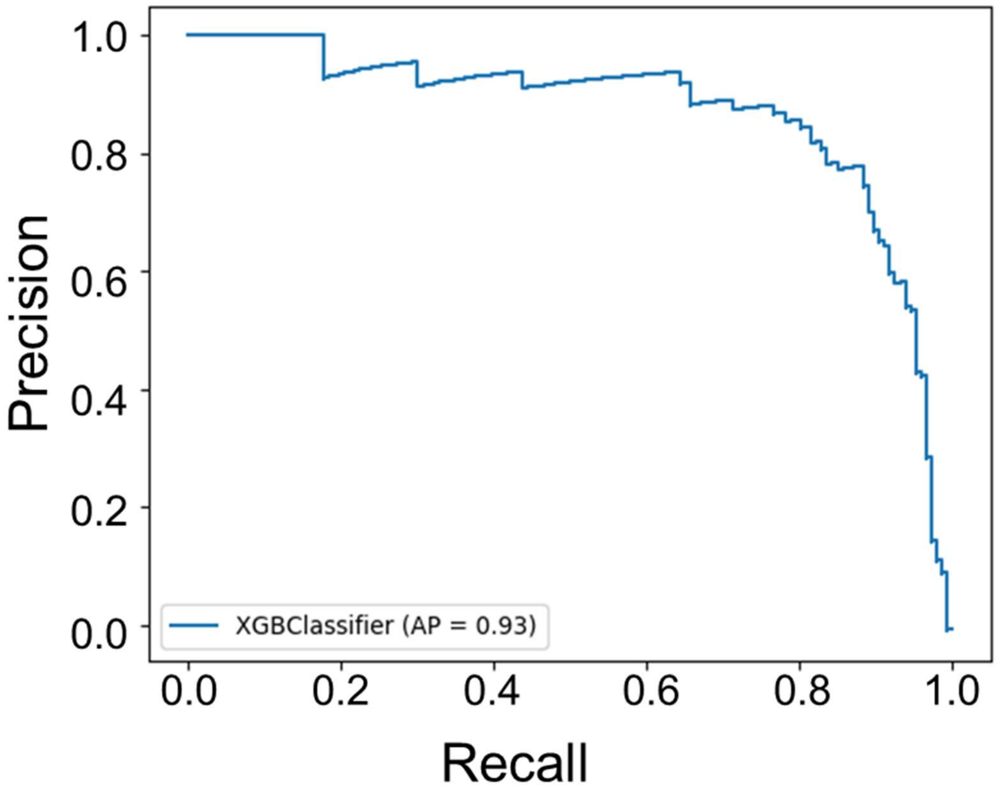

Supplementary Figure 4. Impact of (a) sequential removal of vitals based on feature importance, and (b) including only one vital sign at a time on the predictive model performance. When considering the importance of a feature pertaining to each vital sign in (b), all other vitals were removed from the internal validation set. However, the demographics information was not ablated.

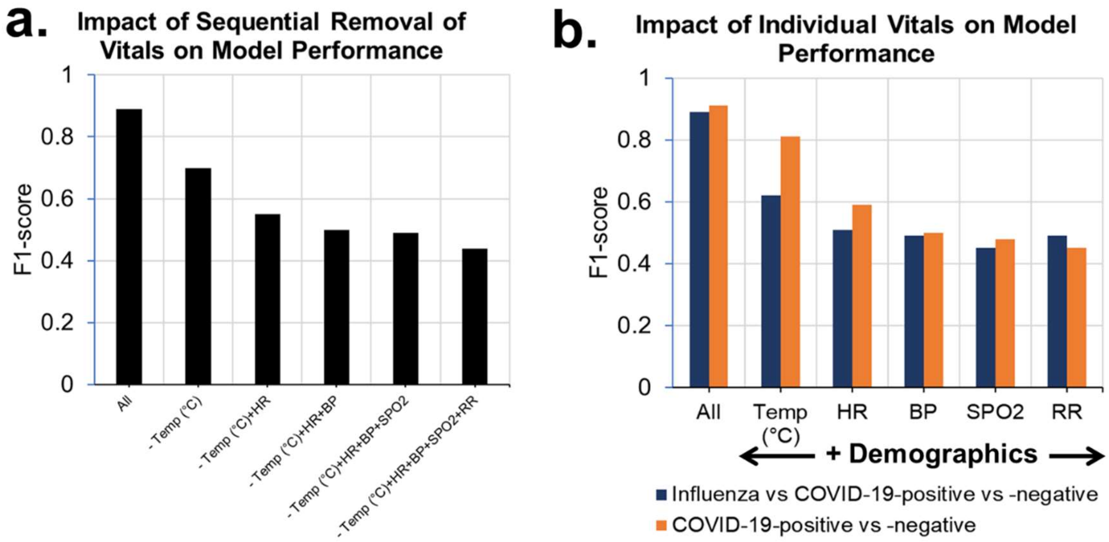

Supplementary Figure 5: Assessment of the performance of different models for the patients presenting to or admitted at the hospital during different seasonal months periods. (a) Receiver Operating Characteristic Curves showing the predictive performance of the influenza versus COVID-19-positive vs COVID-19-neagtive model on the patients grouped on seasonal months. (b) Comparison and evaluation of the performance of four different models for the patient groups based on different seasonal months at identifying Influenza or COVID-19-pos or COVID-19-neg.

a.

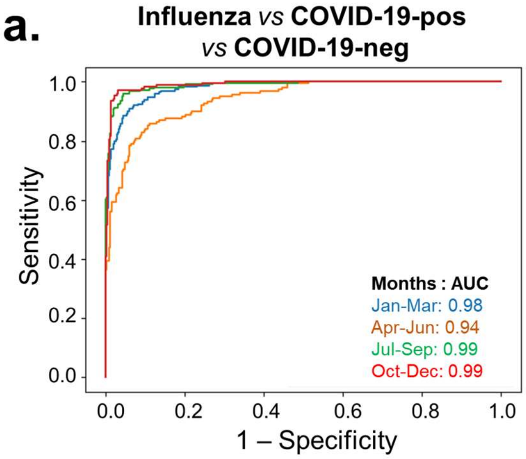

b.

	F1-Score			
**Model Details**	Season1 (Jan-Mar)	Season2 (Apr-Jun)	Season3 (Jul-Sep)	Season4 (Oct-Dec)
**Influenza vs COVID-19-pos vs COVID-19-neg**	0.87	0.79	0.93	0.95
**Influenza *vs* COVID-19-pos**	0.95	0.76	0.95	1
**Influenza *vs* Others**	0.88	0.86	0.99	1
**COVID-19-pos *vs* COVID-19-neg**	0.84	0.93	0.94	0.94

Supplemental Tables.

Supplementary Table 1. Baseline characteristics of the model development/training and model test/internal validation cohort from WVU hospitals

**Attribute Name**	**Train**			**Test**		
	COVID-19-pos	Influenza	COVID-19-neg	COVID-19-pos	Influenza	COVID-19-neg
Patients Count	601	961	1544	146	262	369
Body Mass Index	31.74 ± 8.96	29.75 ± 9.48	29.65 ± 9.33	30.89 ± 8.33	29.58 ± 9.42	29.62 ±10.36
Body Surface Area	2.07 ± 0.35	1.93 ± 0.44	1.96 ± 0.39	2.05 ± 0.35	1.91 ± 0.41	1.96 ± 0.42
Sex						
Male	299	454	740	76	131	176
Female	302	507	804	70	131	193
Ethnicity						
Hispanic or Latino	30	29	14	4	14	3
Not Hispanic or Latino	571	932	1530	142	248	366
Race						
Black or African American	41	77	46	7	18	14
White	513	816	1437	128	221	340
Others	47	68	61	11	23	15
Age Group						
Children and Teens (0 - 20)	16.16 ± 4.52 (55)	11.42 ± 4.57 (236)	12.04 ± 6.09 (105)	15.88 ± 4.52 (8)	11.65 ± 4.02 (75)	9.29 ± 7.22 (18)
Adults (21 - 44)	31.68 ± 7.72 (137)	31.01 ± 6.59 (279)	31.83 ± 6.92 (338)	33.32 ± 7.83 (34)	31.9 ± 6.96 (75)	32.79 ± 6.98 (92)
Older Adults (45 - 64)	55.24 ± 5.9 (178)	54.74 ± 5.86 (222)	55.38 ± 5.76 (406)	55.76 ± 6.28 (38)	54.31 ± 6.02 (60)	54.23 ± 6.12 (98)

Senior Adults (65+)	76.52 ± 8.19 (231)	74.65 ± 7.72 (215)	77.7 ± 8.24 (673)	77.35 ± 8.04 (66)	77.34 ± 7.03 (50)*	77.2 ± 8.1 (154)
Vitals						
Oxygen Saturation (SpO2)	95.64 ± 4.37 (601)	96.67 ± 2.8 (961)	96.28 ± 3.48 (1539)	95.58 ± 3.49 (146)	96.81 ± 3.59 (262)	96.29 ± 5.52 (369)
Body Temperature (Celcius)	36.86 ± 0.6 (601)	37.18 ± 0.71 (961)	36.63 ± 0.45 (1539)	36.85 ± 0.55 (146)	37.15 ± 0.72 (262)	36.66 ± 0.5 (369)
Diastolic BP	75.52 ±13.55 (601)	72.21 ±11.67 (961)	72.42 ± 13.2 (1539)	73.51 ±12.97 (146)	70.41±12.18 (262)	72.95±12.92 (369)
Systolic BP	127.37±18.8 (601)	124.26±19.1 (961)	125.9 ±19.9 (1539)	126.11 ±19.1 (146)	122.74±18.2 (262)	125.93±20.6 (369)
Heart Rate	83.28±15.86 (601)	93.45±19.47 (961)	83.04±16.84 (1539)	80.94 ± 16.2 (146)	94.73±20.07 (262)	85.39±18.48 (367)*
Respiratory Rate	18.53 ± 3.6 (601)	18.85 ± 3.19 (961)	18.21 ± 3.48 (1539)	18.59 ± 3.26 (146)	18.95 ± 3.21 (262)	18.71 ± 9.42 (368)

Supplementary Table 2. Patient demographics of the external validation cohort derived from the TrinetX research network

	Overall	COVID-positive	Influenza	p value
N	3125	1057	2068	
Encounters	15,697	6613	9087	
Sex				0.0001*
Male	1528	568 (53.7)	960 (46.4)	
Female	1597	489 (46.3)	1108 (53.6)	
Ethnicity				0.0001*
Hispanic or Latino	74	41 (3.9)	33 (1.6)	
Not Hispanic or Latino	3051	1016 (96.1)	2035 (98.4)	
Race				<0.0001*
African American	1491	523 (49.5)	968 (46.8)	
White	1254	307 (29.0)	947 (45.8)	
Others	380	227 (21.5)	153 (7.4)	
Overall Age Group	57.15±17.12	57.22±16.64	57.11±17.36	0.87
Children and Teens (0 - 20)	19.27±0.70	18.75±0.50	19.39±0.70	0.10
	22	4 (0.4)	18 (0.9)	
Adults (21 - 44)	33.33±6.73	33.30±6.58	33.35±6.81	0.93
	719	234 (22.1)	485 (23.5)	
Older Adults (45 - 64)	55.88±5.51	55.83±5.57	55.91±5.48	0.80
	1214	435 (41.2)	779 (37.7)	
Senior Adults (65+)	74.47±6.94	74.25±6.78	74.58±7.02	0.45
	1125	373 (35.3)	752 (36.4)	
Vitals				
Oxygen Saturation (SpO2)	95.94±2.49	95.80±2.71	96.08±2.23	0.03*
	1580	796 (50.4)	784 (49.6)	
Body Temperature (°Farenheight)	98.57±1.28	98.55±0.98	98.58±1.46	0.2
	15697	6613 (42.1)	9084 (57.9)	
Diastolic BP	73.09±11.13	73.87±10.72	72.24±11.50	<0.0001*
	9435	4910 (52.0)	4525 (48.0)	
Systolic BP	126.44±18.50	127.10±17.86	125.73±19.15	0.0003*
	9436	4911 (52.0)	4525 (48.0)	
Heart Rate	88.49±15.98	86.94±15.45	89.53±16.25	<0.0001*
	13833	5583 (40.4)	8250 (59.6)	
Respiratory Rate	20.12±4.38	20.89±5.01	19.56±3.76	<0.0001*
	15691	6611 (42.1)	9080 (57.9)	

Values are counts (%) or mean ± standard deviation.
*$p < 0.05$ between COVID-Positive and Influenza group. P values were calculated using independent t test where mean is reported and Chi squared where frequencies are reported.

Supplementary Table 3. Assessment of performance of multi-class classification model at identifying Influenza or COVID-19 amongst patients presenting to or admitted at the hospital in a held-out 20% test set

Performance Measure	Influenza vs Rest	COVID-19-positive vs Rest	COVID-19-negative vs Rest
AUC	0.984	0.972	0.971
Accuracy	0.938	0.946	0.913
F1 Score	0.908	0.844	0.911
Sensitivity (Recall)	0.912	0.781	0.883
Specificity	0.952	0.984	0.943
PPV (Precision)	0.905	0.919	0.940
NPV	0.955	0.951	0.887
FPR	0.048	0.016	0.057
FDR	0.095	0.081	0.060
FNR	0.086	0.219	0.117

Supplementary Table 4. Chief complaint or reason for visit among COVID-positive, -negative and influenza patients

	COVID Positive	Influenza	COVID Negative
Total (%)	747	1223	1913
Respiratory (SOB, Cough, URI/Flu/COVID like symptoms)	339 (51.21)	625 (55.83)	515 (34.85)
Fever	82 (12.39)	218 (18.78)	81 (5.42)
GI (nausea, vomiting, diarrhea, abdominal pain, GI bleed)	60 (9.06)	71 (6.12)	143 (9.57)
Chest pain or other heart problem	24 (3.63)	50 (4.31)	103 (6.89)
CNS (alter mental status, dizziness, syncope, headache, stroke, fall, confusion)	55 (8.31)	45 (3.88)	222 (14.85)
Systemic (generalized fatigue, lethargy, weakness, bodyache, hypo/hyperglycemia lightheadedness, hypotension)	51 (7.70)	59 (5.08)	89 (5.95)
Other (skin problem, urinary problem, pain, injury/trauma, OBGyn problems, ENT problems, medical evaluation, or abnormal tests evaluation, etc.)	51 (7.70)	50 (4.31)	342 (22.88)
Unknown	85 (11.38)	62 (5.07)	418 (21.85)

SOB - Shortness of breath; GI - Gastrointestinal; CNS - Central Nervous System; OBGyn - Obstetrics and Gynecology; ENT - ear, nose, and throat

Supplementary Table 5. Assessment of the performance of Influenza vs COVID-19-positive and Influenza vs Other predictive models at identifying Influenza or COVID-19 amongst patients presenting to or admitted at the hospital in the TriNetX external validation test set. The datasets evaluated below enforces three conditions: (a) no missing values are allowed for body temperature (n=15,697) and (b) no missing values are allowed for both heart rate and body temperature (n=13,834) and (c) no missing values in any of the vital signs (n=1,340)

Performance Measure	Influenza vs COVID-19-positive			Influenza vs Others		
	Body Temperature	Body Temperature + Heart rate	Enforcing no missing Vitals	Body Temperature	Body Temperature + Heart rate	Enforcing no missing vitals
AUC	0.928	0.930	0.949	0.914	0.913	0.954
Accuracy	0.858	0.861	0.883	0.863	0.863	0.881
F1 Score	0.838	0.835	0.869	0.874	0.878	0.892
Sensitivity (Recall)	0.806	0.804	0.907	0.931	0.937	0.932
Specificity	0.901	0.906	0.865	0.791	0.781	0.824
PPV (Precision)	0.872	0.868	0.833	0.824	0.826	0.855
NPV	0.847	0.857	0.926	0.916	0.918	0.912
FPR	0.099	0.094	0.135	0.209	0.219	0.176
FDR	0.128	0.132	0.167	0.176	0.174	0.145
FNR	0.194	0.196	0.093	0.069	0.063	0.067

Supplementary Table 6. Comparison of the performance metrics of the Influenza vs COVID-19-positive XGBoost model trained based on the encounter-month and seasonal-month (Jan-Mar as '1', Apr-Jun as '2', Jul-Sep as '3' and Oct-Dec as '4') on the test set for predicting the given record as COVID-19-positive or influenza.

Performance Measures	Trained on Encounter-month	Trained on Season
AUC	0.988	0.973
Accuracy	0.949	0.912
F1 Score	0.928	0.929
Sensitivity (Recall)	0.925	0.952
Specificity	0.962	0.848
PPV (Precision)	0.932	0.908
NPV	0.958	0.918
FPR	0.038	0.152

Supplementary Methods.

Evaluation Metrics

The following metrics were used to evaluate the performance of various models developed as part of this study. One of the most determined statistical measures is Sensitivity (also known as recall, hit rate or true positive rate TPR). Sensitivity measures the proportion of actual positives that are correctly identified as positives: TP / (TP + FN). Specificity, also known as selectivity or true negative rate (TNR), measures the proportion of actual negatives that are correctly identified as negatives: TN / (FP + TN). The Positive Predictive Value (PPV = TP / (TP + FP)), also known as Precision and the Negative Predictive Value (NPV = TN / (TN + FN)) are measures of the performance of a diagnostic test. The False Positive Rate (FPR = FP / (FP + TN)) or fall-out is the ratio between the number of negative events incorrectly categorized as positive (false positives) and the total number of actual negative events (regardless of classification). The False Negative Rate (FNR= FN / (FN + TP)) measures the proportion of the individuals where a condition is present for which the test result is negative. Accuracy (ACC) is a measure of all correctly predicted observations and defined as (TP + TN) / (TP + TN + FP + FN). The F1 Score is a measure of a test’s accuracy, defined as the harmonic mean of precision and recall i.e., 2TP / (2TP + FP + FN).
